# Supplementary material for: Effect of Morphological Characteristics and Biomineralization of 3D-Printed Gelatin/Hyaluronic Acid/Hydroxyapatite Composite Scaffolds on Bone Tissue Regeneration
Source: Int J Mol Sci. 2021 Jun 24;22(13):6794. doi: 10.3390/ijms22136794 (PMC8267715; doi:10.3390/ijms22136794)
Supplement: Supplementary file 1 [file ijms-22-06794-s001.zip › ijms-1256516-supplementary.pdf]

Supplementary Information

Effect of Morphological Characteristics and Biomineralization of 3D-Printed Gelatin/Hyaluronic acid/Hydroxyapatite Composite Scaffolds on Bone Tissue Regeneration

Jae-Woo Kim, Yoon Soo Han, Hyun Mee Lee, Jin-Kyung Kim, and Young-Jin Kim*

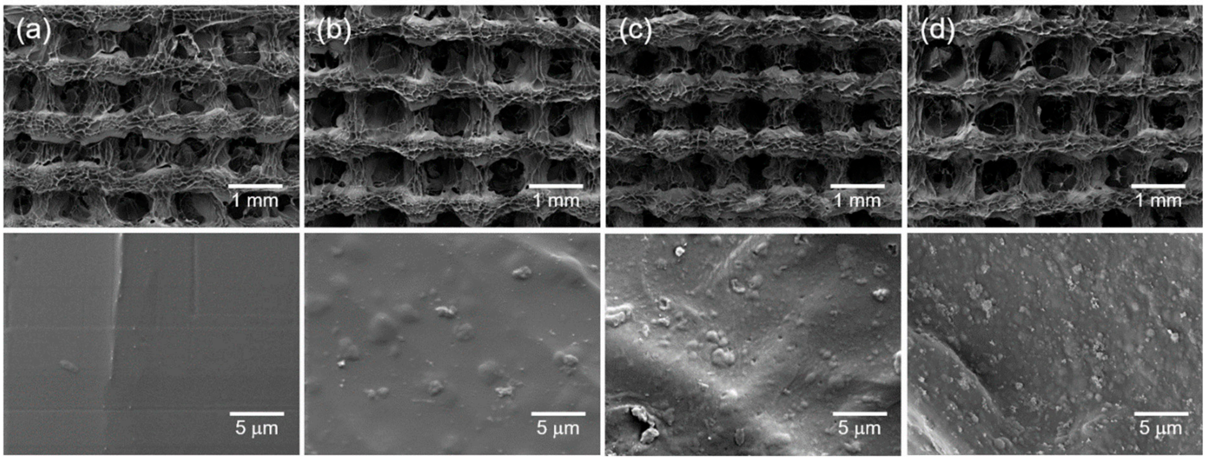

Figure S1. SEM images of (a) GEHA0, (b) GEHA10, (c) GEHA20, and (d) GEHA40 composite scaffolds.

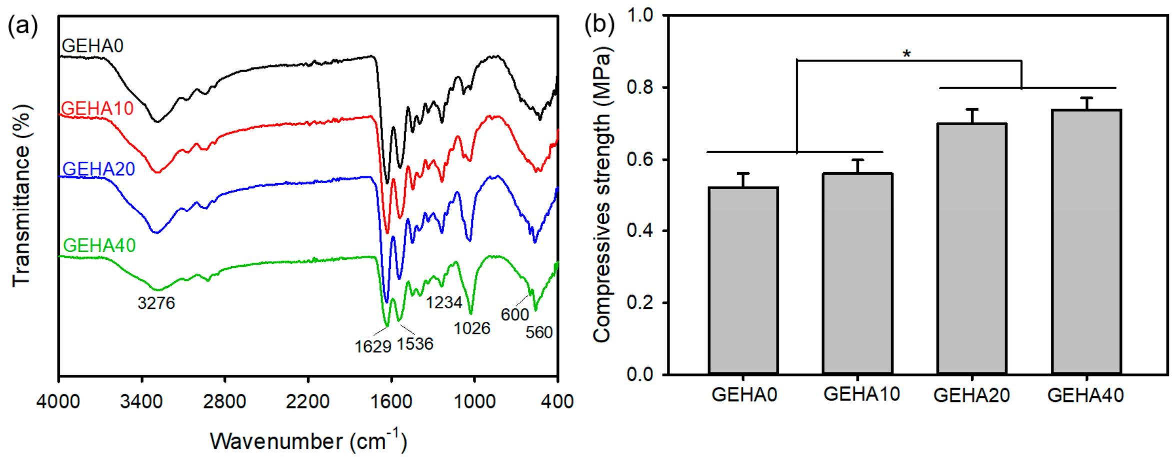

Figure S2. (a) FTIR spectra and (b) compressive strength of composite scaffolds containing different amount of HAp nanoparticles ($n = 4$).
